# Weight Loss by Diet Versus Metabolic Surgery Increases Circulating NT-proANP in Obese Individuals

**DOI:** 10.3390/jcm15041515

**Published:** 2026-02-14

**Authors:** Andreas Schmid, Maria Koukou, Thomas Karrasch, Andreas Schäffler

**Affiliations:** Department of Internal Medicine III, Giessen University Hospital, 35392 Giessen, Germany

**Keywords:** NT-proANP, NPRA, obesity, adipose tissue, Roux-en-Y gastric bypass (RYGB), low-calorie formula diet (LCD)

## Abstract

**Background:** Natriuretic peptides are endocrine factors that regulate various physiological processes via natriuretic peptide receptors (NPRs). Regulation of the atrial natriuretic peptide ANP during weight loss remains widely unknown. The present study investigated serum quantities of the circulating ANP precursor NT-proANP in obesity and during therapy-induced weight loss. **Methods:** The study enrolled 284 severely obese individuals. A total of 163 patients underwent metabolic surgery (either Roux-en-Y gastric bypass or vertical sleeve gastrectomy) and 121 patients participated in a non-invasive obesity therapy applying low-calorie formula diet. Anthropometric and physiological data were assessed, and blood serum was prepared at study baseline and at follow-up visits (3 and 12 months after start of intervention). Subcutaneous and visceral adipose tissue specimen were obtained from metabolic surgery patients. Circulating NT-proANP levels were determined by ELISA and gene expression levels of the receptor NPRA in adipose tissue were quantified by real-time RT-PCR. **Results:** Comparative analysis revealed significantly higher NPRA expression in visceral than in subcutaneous adipose tissue. NT-proANP levels significantly increased during weight loss over 12 months upon diet and metabolic surgery. NT-proANP serum concentrations were positively correlated with fibroblast growth factors 19 and 21 quantities at study baseline and considerably increased during weight loss in both cohorts after 12 months. We conclude that weight loss is a positive regulator of circulating NT-proANP quantities, regardless of the applied therapy.

## 1. Introduction

The family of natriuretic peptides (NPs) comprises atrial (ANP), brain (BNP), and C-type NP (CNP), all of them sharing a similar molecular structure and analogous way of processing and maturation [1]. After proteolytic cleavage of an N-terminal signal peptide from the respective pre-pro-hormones in order to enable secretion, the secreted pro-hormones are further processed by extracellular cleavage, resulting in an inactive N-terminal peptide and a bioactive C-terminal peptide [2]. Natriuretic peptides exert diverse physiological functions. While BNP acts as a protective antagonist of ventricular fibrosis and CNP is involved in bone metabolism, ANP represents an important vasorelaxant agent and regulator of blood pressure homeostasis [1]. ANP-induced relaxation of smooth muscle cells is mediated by the guanylyl cyclase NP receptor (NPR)A [3] via cGMP-dependent protein kinases [4]. ANP increases the permeability of vascular endothelium in order to decrease blood pressure [5].

Since adipose tissue and adipocyte cell lines exhibit high expression levels of NPRA, NPRB, and PRC [6,7] that are regulated differentially during adipocyte differentiation, it is reasonable to assume that this organ is responsive to a variety of NP-induced effects such as lipolysis. Obesity and metabolic syndrome are characterized by an upregulation of the endopeptidase neprilysin catalyzing cleavage and degradation of multiple peptides including ANP [8]. Thus, one could assume that circulating ANP quantities become downregulated in obese individuals. Furthermore, circulating NT-proANP levels have been reported to be negatively correlated with insulin resistance and insulin administration [9]. Of particular interest, systemic BNP levels have been reported to decrease in obesity and to recover during weight loss induced by surgical therapy [10]. Due to the present state of the literature, the involvement of metabolic factors in the regulation of circulating BNP has been studied more extensively than it is the case with ANP. Knowledge concerning the role of ANP/NPRA action in obesity remains unsatisfying so far, at least in the human system. This includes the regulation of local NPRA gene expression levels in different adipose tissue compartments as well as systemic ANP concentrations under the conditions of obesity and during therapeutically induced weight loss.

The present study in severely obese individuals investigated

- NPRA gene expression in subcutaneous and visceral adipose tissue compartments of obese subjects.
- NT-proANP serum levels of obese individuals and their correlations with anthropometric and biochemical parameters.
- Kinetics of NT-proANP serum concentrations during weight loss induced by either low-calorie diet or metabolic surgery.

## 2. Materials and Methods

### 2.1. Study Population and Data Collection

Human serum samples and adipose tissue specimen were obtained within the ROBS (Research in Obesity and Bariatric Surgery) study which has been introduced previously [11]. Briefly, as an open-label, non-randomized, monocentric, prospective, and observational study, ROBS enrolled severely obese patients routinely undergoing weight loss therapy at the University Hospital Giessen, Germany, either by bariatric surgery (BS) (VSG, vertical sleeve gastrectomy; RYGB, Roux-en-Y gastric bypass surgery) or by low-calorie formula diet (LCD). The study was approved by the local ethical committee at the University of Giessen, Germany (file code AZ 101/14), and all patients gave their informed consent. Data anonymization and privacy policies were applied accurately. The study examined adult individuals (age ≥ 18 years) with severe obesity. Inclusion criterium for participation in the LCD program was a minimum BMI of 30 kg/m^2^ while allocation of patients to bariatric surgery required either a BMI ≥ 40 kg/m^2^ or BMI ≥ 35 kg/m^2^ with coexisting manifested diabetes mellitus [11]. General exclusion criteria for the study were pregnancy, neoplasm, severe psychiatric disorders, psychosis and psychopathologic instability, untreated bulimia nervosa and binge eating behavior, evidence of or suspicion on underlying endocrine diseases, and use of illicit drugs.

The applied annual LCD program (OPTIFAST^®^ 52, Nestle Health Science, Frankfurt am Main, Germany) started with an initial fasting period (12 weeks) during which food intake was restricted to 865 kilocalories (kcal) per day by using an exclusive and liquid formula diet (OPTIFAST Professional, Nestle Health Science, Frankfurt am Main, Germany). The dietary formulation contained minerals, vitamins, and the following ingredients/macronutrients (per 100 g): 392 kcal, 11 g fat (1.2 g saturated fatty acids (FAs), 5.0 g monounsaturated FA, and 3.8 g polyunsaturated FA), 34 g carbohydrates, 36 g protein (mainly milk protein), 6.5 g fibers, 1.2 g salt, and 17 g lactose. The patients received 5 meals per day of 44 g formulated nutritional powder dissolved in 160 mL H_2_O. During the following transitional phase (8 weeks), there was a stepwise conversion to a healthy, balanced mixed diet (flexible carbohydrates, fat-restricted), with subsequently decreasing proportions of liquid diet. The final period of 31 weeks stabilized the adjusted nutritional behavior without formula diet.

In the context of regular group meetings, LCD subjects participated in physical exercise therapy based on their individual fitness level at study baseline. Exercise involved stretching as well as endurance, mobility, coordination, and strength training.

The present study analyzed data from a total of 284 ROBS subjects (121 in the LCD and 163 in the BS group) having completed the study time-points V0 (baseline). A total of 132 BS patients received RYGB and 31 underwent VSG. A total of 124 individuals (55 in LCD and 69 in BS group) further completed V3 and V12 (3- and 12-month follow-up, respectively). The performed analyses and presented data represent an extension of the study initially introduced by Brock et al. in 2019 [11]. Data collection was performed prior to metabolic surgery or start of dietary intervention (V0), 3–5 days post-surgery (V1; exclusively for BS patients), and after 3 and 12 months (V3 and V12). The examination of the patients included an anthropometric assessment, collection of clinical and psychological data as well as medication, smoking habits, and nutritional status, routine laboratory examination, and protein quantification in blood serum samples. The overall characteristics of the study cohort have been published previously [12]. The present study investigated a subset of patients with available data on serum NT-proANP levels. Baseline (V0) anthropometric and metabolic characteristics are depicted in Supplementary Table S1.

### 2.2. Gene Expression Analysis in Human Adipose Tissue

Nucleic acid extraction from human subcutaneous and visceral adipose tissue was performed with TRIzol reagent (Thermo Fisher, Braunschweig, Germany) and chloroform (Sigma-Aldrich, Deisenhofen, Germany) prior to subsequent RNA isolation applying the RNeasy^®^ Mini Kit (Qiagen, Hilden, Germany). Reverse transcription of RNA (QuantiTect Reverse Transcription Kit from Qiagen, Hilden, Germany) was performed in order to generate the corresponding cDNA for quantitative real-time PCR (qRT-PCR) (iTaq Universal SYBR Green Supermix, CFX Connect RT-PCR system; Bio-Rad, Munich, Germany). Target gene mRNA levels were quantified using the following primer sequences:

Human NPRA: 5′-GGAGATTGCCCTGAGGAG-3′/5′-TTGCTGCTGTTCTCCCTGTTA-3′.

Human GAPDH: 5′-GAGTCCACTGGCGTCTTCAC-3′/5′-CCAGGGGTGCTAAGCAGTT-3′.

All applied oligonucleotides were purchased from Metabion (Martinsried, Germany). Expression levels were quantified applying the ddC_T_ method with normalization to expression of murine GAPDH.

### 2.3. Enzyme-Linked Immunosorbent Assay (ELISA)

NT-proANP concentrations in blood serum were determined applying an ELISA Kit (human NT-proANP DuoSet Development Kit, R&D Systems; Minneapolis, MN, USA) with a detection range of 0.3–20 ng/mL. Measurements were performed in technical duplicates and were repeated in cases of CV exceeding 20%.

### 2.4. Statistical Analysis

Statistical analysis was performed applying the software package SPSS (Version 29.0; IBM, Armonk, NY, USA). Unrelated groups were compared by Mann–Whitney U-test (*n* = 2 groups). Comparison of related samples was performed by Friedman test (*n* > 2 groups). Bonferroni correction was applied for multiple comparisons. Data regarding comparisons between groups are graphically presented as bar diagrams (means ± standard error of the mean). Spearman’s rank correlation coefficient was used to evaluate associations between variables. These data are presented as scattered plots. In the text, data are given as means ± standard deviation. Case numbers are depicted in the respective figures. In general, a *p* value < 0.05 was considered statistically significant.

## 3. Results

### 3.1. Basal NT-proANP Serum Quantities in Severely Obese Individuals

**Figure 1:**
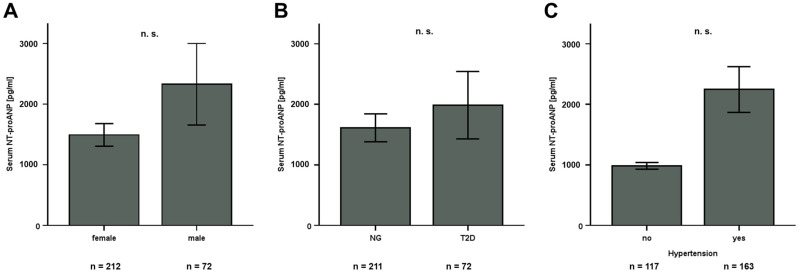
Subgroup analysis of NT-proANP serum levels in the entire study cohort (*n* = 284). NT-proANP quantities do not significantly differ between males and females (**A**), normoglycemic (NG) and diabetic patients (**B**), and normotensive and hypertensive individuals (**C**). Bar graphs display mean values ± SEM (standard error of the mean). Mann–Whitney U-test was applied for comparison of unrelated samples. n.s., not significant.

The overall characteristics of the entire ROBS study cohort including general anthropometric and biochemical parameters have been published previously [10,11]. The current study examined a subset of 284 patients, including 121 individuals undergoing LCD (39 males, 82 females; mean BMI at V0: 43.4 ± 5.9 kg/m^2^) and 163 subjects undergoing bariatric surgery (33 males, 130 females; mean BMI at V0: 53.21 ± 6.99 kg/m^2^). At study baseline, NT-proANP serum concentrations were 1542 ± 2820 pg/mL in the LCD and 1867 ± 4157 pg/mL in the bariatric surgery sub-cohort. No significant sexual dimorphism was observed for both subgroups as well as for the whole study cohort (Figure 1A). Further subgroup analysis revealed no significant impact of type 2 diabetes mellitus or hypertonic blood pressure on NT-proANP quantities (Figure 1B,C).

**Figure 2:**
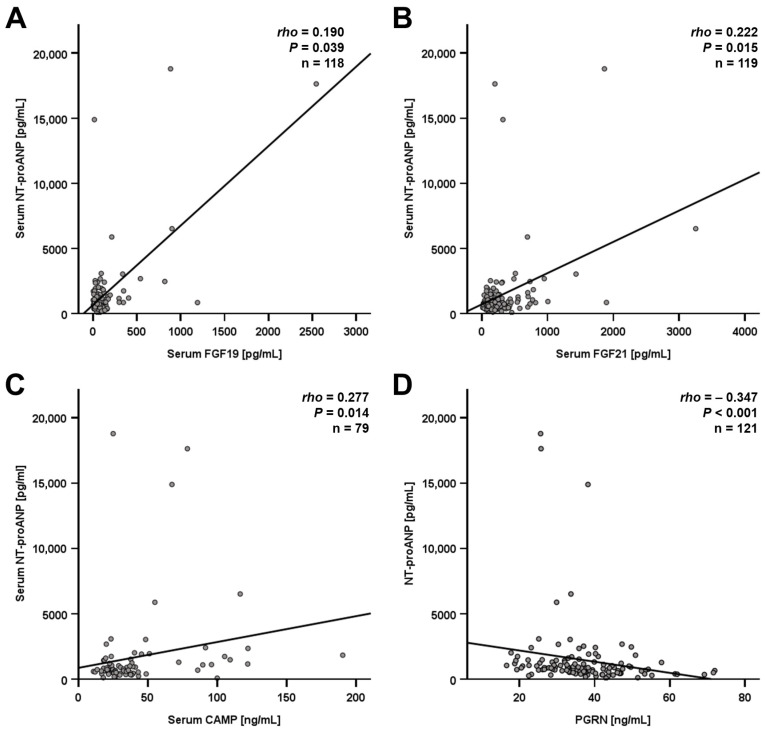
Correlations of NT-proANP serum levels with circulating protein concentrations among LCD patients (*n* = 121). NT-proANP quantities correlate positively with fibroblast growth factor (FGF) 19/21 (**A**,**B**) and cathelicidin antimicrobial peptide (CAMP) (**C**) but negatively with progranulin (PGRN) (**D**). Spearman’s rank correlation coefficient was applied. rho, correlation coefficient; LCD, low calorie diet.

Correlation analysis was applied in order to identify potential co-regulations or interactions of NT-proANP levels with adipokines measured earlier in this cohort. Among LCD participants, significant positive correlations of NT-proANP quantities with serum concentrations of FGF-19, FGF-21, and cathelicidin antimicrobial peptide (CAMP/LL37) were detected (Figure 2A–C). Furthermore, NT-proANP correlated negatively with systemic progranulin (PGRN) quantities (Figure 2D).

**Figure 3:**
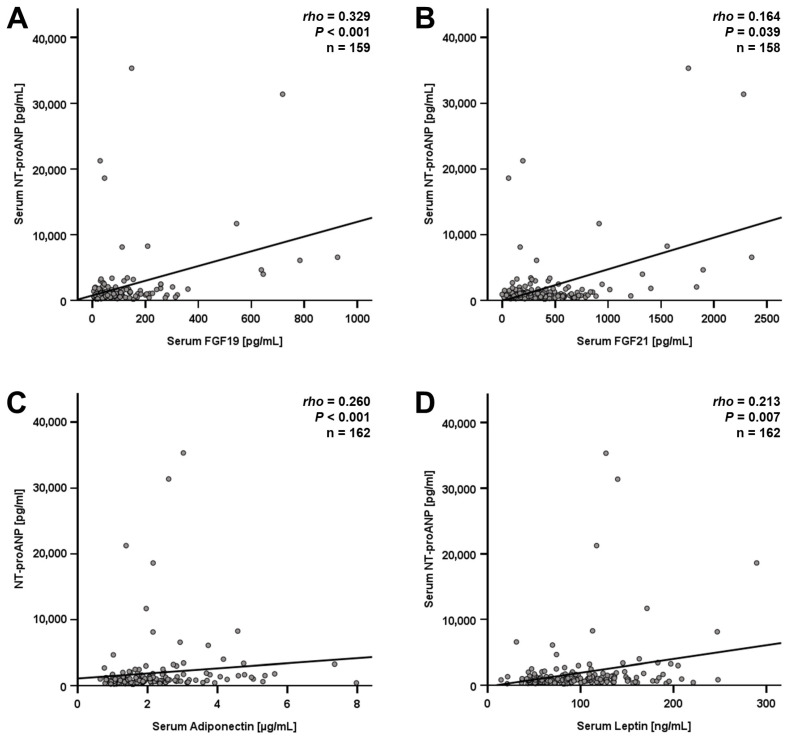
Correlations of NT-proANP serum levels with circulating protein concentrations among BS patients (*n* = 163). NT-proANP quantities correlate positively with fibroblast growth factor (FGF) 19/21 (**A**,**B**), adiponectin (**C**), and leptin (**D**). Spearman’s rank correlation coefficient was applied. rho, correlation coefficient; BS, bariatric surgery.

Similar to the findings within the LCD cohort, NT-proANP correlated positively with FGF-19 and -21 concentrations among BS patients (Figure 3A,B). Additional positive correlations were detected for adiponectin and leptin quantities (Figure 3C,D). Although these correlations mentioned above were statistically significant, the respective correlation coefficients (rho) were rather weak, questioning the physiological relevance of these findings. Immunomodulatory adipokines substantially involved in obesity such as C1q/TNF-related protein 3 (CTRP3) and meteorin-like (Metrnl) [13,14] did not significantly correlate with NT-proANP quantities, neither among LCD participants (*n* = 121; CTRP3: *rho* = −0.151, *p* = 0.097; and Metrnl: *rho* = −0.032, *p* = 0.724) nor among bariatric patients (*n* = 163; CTRP3: *rho* = −0.002, *p* = 0.979; and Metrnl: *rho* = 0.105, *p* = 0.184).

### 3.2. Natriuretic Peptide Receptor NPRA Expression in Subcutaneous and Visceral Adipose Tissue

**Figure 4:**
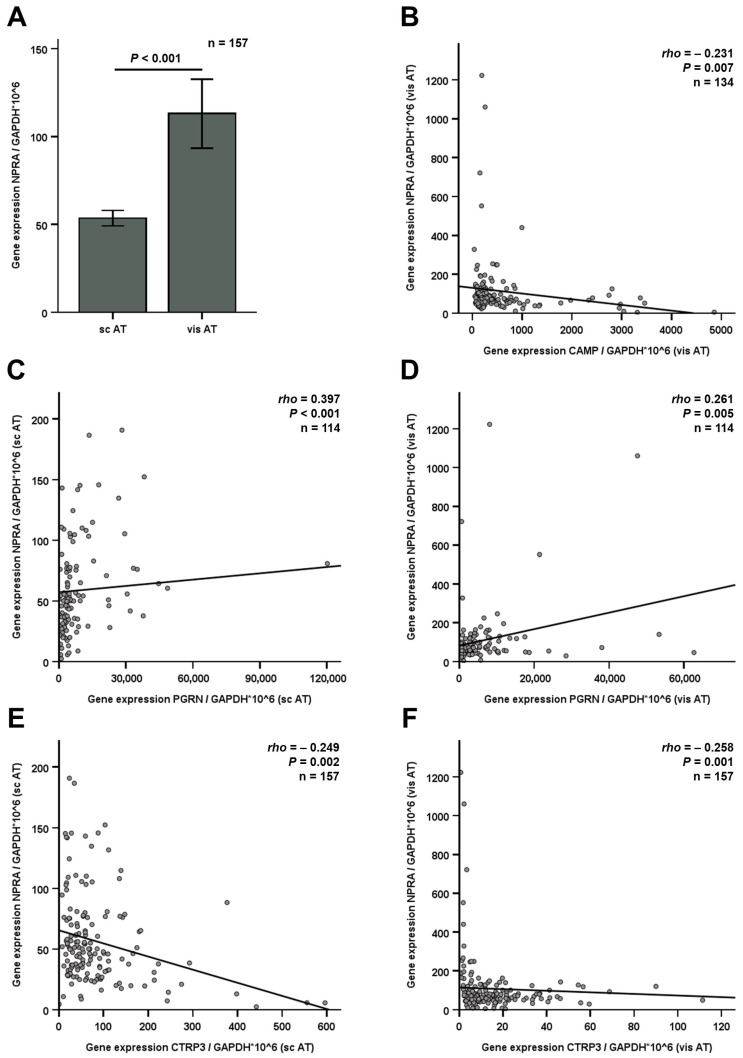
Correlations of NPRA and adipokine gene expression levels in subcutaneous (sc AT) and visceral adipose tissue (vis AT) of BS patients (*n* = 157). The expression of NPRA is significantly higher in visceral adipose tissue when compared to subcutaneous (abdominal) adipose tissue of obese patients (**A**). Positive correlations of natriuretic peptide receptor A (NPRA) mRNA concentrations with cathelicidin antimicrobial peptide (CAMP) (**B**), progranulin (PGRN) (**C**,**D**), and C1q/TNF-related protein 3 (CTRP3) (**E**,**F**) expression levels are depicted. Spearman’s rank correlation coefficient was applied. rho, correlation coefficient; vis, visceral; sc, subcutaneous; AT, adipose tissue.

Specimen from subcutaneous and visceral adipose tissue of obese individuals were obtained during metabolic surgery (*n* = 157, paired samples). After initial shock-freezing and cryo-storage, mRNA was isolated for gene expression analysis via RT-PCR methods. Expression levels of the natriuretic peptide receptor NPRA were investigated in both adipose tissue locations and were observed to be unaffected by age, sex, type 2 diabetes mellitus, and hypertension. Interestingly, the expression of NPRA in visceral adipose tissue was significantly (*p* < 0.001) higher than in subcutaneous adipose tissue in obese patients (Figure 4A). This highly significant result in a large cohort (with *n* = 157 paired tissue samples) confirms the trend of a smaller subset (*n* = 44) in a previous study [7]. NPRA and CAMP mRNA concentrations were negatively correlated in visceral adipose tissue (Figure 4B). Furthermore, positive correlations with PGRN and negative correlations with CTRP3 gene expression levels were detected in both adipose tissue locations (Figure 4C–F).

### 3.3. Increase in NT-proANP Concentrations During Weight Loss

**Figure 5:**
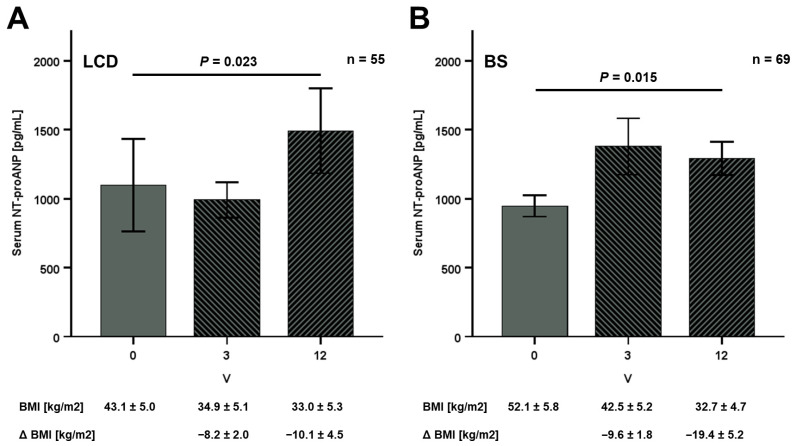
Kinetics of NT-proANP serum levels during weight loss therapy. NT-proANP serum concentrations increase during 12 months of weight loss, induced either by low calorie diet (LCD, *n* = 55) (**A**) or by metabolic surgery (BS, *n* = 69) (**B**). Mean BMI levels and the decrease in BMI (Δ BMI) are given at V0, V3, and V12 (months), and the Friedman test was applied for comparison of related samples.

In both study sub-cohorts, therapeutical intervention resulted in substantial metabolic improvement, including highly significant weight loss, BMI decline, and body fat reduction within 12 months, as reported previously [11]. A total of 124 individuals (55 LCD, 69 BS) completed all follow-up visits within the study period and were examined regarding systemic NT-proANP kinetics. LCD did not significantly affect NT-proANP levels within the initial 3 months but finally resulted in an approximately 40% increase at V12 (Figure 5A). Long-term therapeutical success was accompanied by a significant rise in NT-proANP serum concentrations of ~40% in the first 3 months. These quantities remained elevated at an equal level after 12 months, indicating a long-term up-regulation of systemic NT-proANP levels (Figure 5B).

## 4. Discussion

The present study investigated circulating NT-proANP quantities in a large and well-characterized cohort of severely obese individuals undergoing either conservative (low calorie diet) or surgical therapy (metabolic surgery). The present longitudinal data on systemic NT-proANP kinetics during weight loss have not yet been documented in a large study cohort. In a small clinical cohort of obese individuals undergoing gastric bypass surgery, NT-proANP serum levels increased 6 months after surgery [15]. Furthermore, mid-regional pro-atrial natriuretic peptide (MR-proANP) quantities have been found to substantially increase and remain elevated within 12 months after RYGB surgery in hypertensive obese patients [16]. Obesity might be mechanistically linked to low natriuretic peptide levels in humans [17] due to four observations. First, NT-proANP induces lipolysis in adipocytes by interaction with natriuretic peptide receptor A (NPRA) [18]. Second, this lipolytic effect is decreased in obesity [19]. Third, decreased NPRA expression levels in adipose tissue are associated with obesity and related metabolic complications such as insulin resistance and type 2 diabetes mellitus [20]. Fourth, oral lipid ingestion in vivo significantly lowers serum NT-proANP concentrations [7]. The present data documenting the significant NT-proANP increase during weight loss independent of the therapeutical approach fit very well with the observations mentioned above and might indicate the mechanism by which caloric restriction per se induces lipolysis and thus, weight loss. It has been known for decades that the visceral adipose tissue is characterized by a higher lipolytic activity when compared to subcutaneous adipose tissue. We could demonstrate a significant higher expression of NPRA in visceral adipose tissue suggesting that ANP-induced lipolysis represents an important pathway in addition to basal and catecholamine/β3 adrenergic receptor-mediated lipolysis.

**Figure 6:**
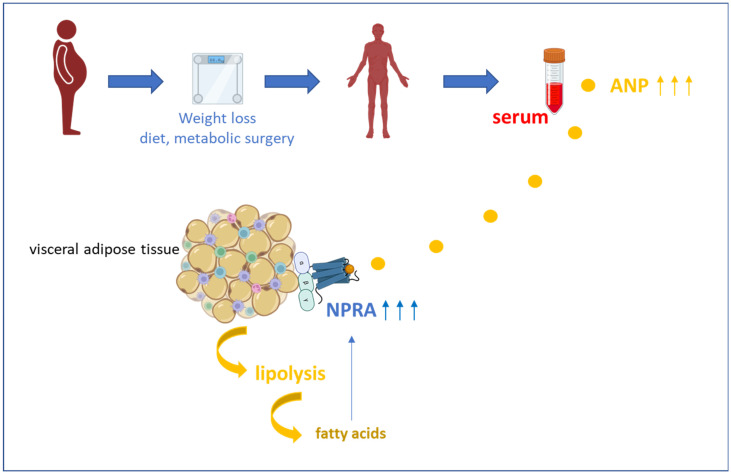
The concept of ANP/NPRA-mediated lipolysis during weight loss in obesity. Caloric restriction induced by diet or metabolic surgery results in a chronic elevation of serum NT-proANP concentrations (present data). Since the visceral adipose tissue is characterized by high NPRA expression levels (present data), this mechanism could represent a potent mechanism of lipolysis-induced weight loss upon diet or metabolic surgery. As demonstrated previously [7], certain fatty acids liberated by adipocyte lipolysis are able to upregulate NPRA expression in adipocytes. Conceptually, these mechanisms could build a positive feedback loop during long-term weight loss. ANP, atrial natriuretic peptide; NPRA, natriuretic peptide receptor A.

In the present study cohort, no significant effects of sex or metabolic dysregulation (type 2 diabetes, dyslipidemia, and hypertension) on circulating NT-proANP levels or on adipose tissue NPRA mRNA quantities were detected. In earlier studies, we could demonstrate in vitro in adipocytes that NPR-A mRNA expression was significantly upregulated by oleic acid and linoleic acid whereas margarinic acid, myristic acid, eicosapentaenoic acid, and docosahexaenoic acid significantly reduced NPR-A mRNA expression [7]. Taken together, caloric restriction by diet or metabolic surgery could result in weight loss by induction of ANP/NPRA-mediated lipolysis, especially in visceral adipose tissue. As a consequence, the local release of fatty acids such as oleic acid from adipocytes might co-stimulate NPRA expression as part of a self-reinforcing, positive feedback loop in weight loss-mediated lipolysis (Figure 6).

Correlation analysis revealed substantial positive correlations of circulating NT-proANP and fibroblast growth factor (FGF19/21) quantities. This observation is consistent with the findings we reported in a previous study on metabolically healthy, predominantly normal-weight individuals [7]. Taken together, data from both studies suggest the existence of an obesity-independent correlation of NT-proANP concentrations with FGF-19 and FGF-21 quantities. Since natriuretic peptides and FGF-21 have been considered as cardio-myokines being involved in lipid metabolism, adipocyte browning mechanisms, and thermogenesis in adipose tissue [21], a putative coregulation of their systemic levels might be of high relevance for these physiological processes and should motivate future research on this issue.

Our observed correlations of NT-proANP with adipokines (adiponectin, leptin, progranulin, and cathelicidin antimicrobial peptide) were not detected consistently in both clinical sub-cohorts (LCD and BS) and the respective correlations coefficients were very weak questioning physiological relevance. Since the two groups significantly differ in terms of BMI, these correlations might substantially depend on the extent of obesity and/or associated parameters. Future extensive research, involving in vitro/ex vivo settings in adipocyte culture, will be needed in order to further elucidate a potential regulatory link between systemic ANP, secretion levels of these adipokines, and adipose inflammation.

In contrast to NT-proANP, NT-proBNP regulation in obesity and during weight loss has been investigated more extensively so far. Previous studies reported predominantly elevated circulating levels after bariatric surgery [10,15,22] and are in accordance with reports from a recent systematic review by Wong et al. [23], whereas Hollstein et al. detected no significant changes neither by surgical nor by dietary intervention [24].

Our findings imply that weight loss and caloric restriction per se might represent a positive and long-term regulator of circulating NT-proANP concentrations independent of the therapeutical approach.

Of course, there are several limitations of the present study. Its primary interest was the longitudinal investigation of molecular parameters—including innovative parameters such as systemic bile acids [25] and immunomodulatory adipokines [13,14,26]—whereas some other examinations, e.g., assessment of pulmonary capacity and adipose tissue tomography, were not covered by the study design due to constraints regarding logistics and scientific focus.

The presented data are of primarily descriptive nature, and the documented correlations are rather weak. The biological relevance of these findings therefore remains somewhat debatable. Furthermore, a direct mechanistic association between local expression levels of the receptor NPRA, peripheral NT-proANP quantities, and impact on adipocyte physiology cannot be postulated at this point. Further elucidation of this important issue requires future research applying detailed in vitro studies. Importantly, the described long-term kinetics of NT-pro-ANP levels in these large and well-characterized study cohorts represent a novel finding. Therefore, future clinical research should be encouraged by the present findings in order to further elucidate a mechanistic role of the NT-pro-ANP/NPRA system in obesity, weight loss, lipolysis, and adipocyte physiology.

## 5. Conclusions

We report a highly significant increase in NT-proANP serum concentrations during therapy-induced weight loss by low calorie diet and metabolic surgery. The observed effect was not particularly specific for one of the applied weight loss strategies. Preceding the long-term increase, surgical patients exhibited a transient decline of serum NT-proANP quantities within the very early post-surgical days when body weight has not yet changed. The ANP/NPRA system is a potential candidate for weight loss-induced lipolysis, especially in visceral adipose tissue.

## Data Availability

The data presented in this study are available on reasonable request from the corresponding author.

## Supplementary Materials

The following supporting information can be downloaded at https://www.mdpi.com/article/10.3390/jcm15041515/s1, Table S1: Baseline (V0) anthropometric and metabolic characteristics of patients participating in the ROBS study. Data are presented as means ± standard deviation. BMI, body mass index; CRP, c-reactive protein; HbA1c, hemoglobin A1C; HDL, high-density lipoprotein; LDL, low-density lipoprotein; WHR, waist-hip ratio.
