# Supplementary material for: Weight Loss by Diet Versus Metabolic Surgery Increases Circulating NT-proANP in Obese Individuals
Source: J Clin Med. 2026 Feb 14;15(4):1515. doi: 10.3390/jcm15041515 (PMC12942638; doi:10.3390/jcm15041515)
Supplement: Supplementary file 1 [file jcm-15-01515-s001.zip › jcm-4085581-supplementary.pdf]

Parameters V0	Low calorie diet (n = 121)	Bariatric surgery (n = 163)
Sex	82 female (67.8 %)<br>39 male (32.2 %)	130 female (79.8 %)<br>33 male (20.2 %)
Age (years)	41.8 ± 12.1	39.9 ± 11.2
Body weight (kg)	128.5 ± 22.1	153.1 ± 26.2
BMI (kg/m^2)	43.4 ± 5.9	53.2 ± 7.0
WHR	0.95 ± 0.13	0.95 ± 0.12
HbA_{1c} (%)	5.7 ± 0.7	6.1 ± 1.0
Total cholesterol (mg/dL)	190 ± 36	182 ± 36
LDL cholesterol (mg/dL)	132 ± 33	123 ± 39
HDL cholesterol (mg/dL)	48 ± 13	46 ± 11
Triglycerides (mg/dL)	140 ± 68	149 ± 88
CRP (mg/dL)	11.0 ± 24.6	13.7 ± 12.4

Supplementary Table 1: Baseline (V0) anthropometric and metabolic characteristics of patients participating in the ROBS study. Data are presented as means ± standard deviation. BMI, body mass index; CRP, c-reactive protein; HbA_{1c}, hemoglobin A1C; HDL, high-density lipoprotein; LDL, low-density lipoprotein; WHR, waist-hip ratio
